# Development of the Tumor-Specific Antigen-Derived Synthetic Peptides as Potential Candidates for Targeting Breast and Other Possible Human Carcinomas

**DOI:** 10.3390/molecules24173142

**Published:** 2019-08-29

**Authors:** Subhani M. Okarvi, Ibrahim AlJammaz

**Affiliations:** Cyclotron and Radiopharmaceuticals Department, King Faisal Specialist Hospital and Research Centre, P.O. Box 3354, Riyadh 11211, Saudi Arabia

**Keywords:** Tumor-associated antigens, HER2, MUC1, Breast cancer, Molecular imaging, Synthetic peptides

## Abstract

The human epidermal growth factor receptor 2 (HER2) represents one of the most studied tumor-associated antigens for cancer immunotherapy. The receptors for HER2 are overexpressed in various human cancers, such as breast and ovarian cancer. The relatively low expression of this antigen on normal tissues makes it a clinically useful molecular target for tumor imaging and targeted therapy. HER2 overexpression is correlated with aggressive tumor behavior and poor clinical outcomes. Thus, HER2 has become an important prognostic and predictive factor, as well as a potential molecular target. Due to the heterogeneity of breast cancer and possible discordance in HER2 status between primary tumors and distant metastases, assessment of HER2 expression by noninvasive imaging is important. Molecular imaging of HER2 expression may provide essential prognostic and predictive information concerning disseminated cancer and aid in the selection of an optimal therapy. Another tumor-specific antigen is MUC1, which is silent on normal tissues, but overexpressed in almost all human epithelial cell cancers, including >90% of human breast, ovarian, pancreatic, colorectal, lung, prostate, and gastric cancers and is a promising tumor antigen with diagnostic as well as the therapeutic potential of cancer. Radiolabeled small peptide ligands are attractive as probes for molecular imaging, as they reach and bind the target receptor efficiently and clear from blood and non-target organs faster than bulky antibodies. In this study, HER2 and MUC1-based peptides were synthesized and preclinically evaluated in an effort to develop peptide-based SPECT radiopharmaceuticals derived from tumor-associated antigens for the detection of breast cancer. Our findings demonstrate that the tumor antigen peptides radiolabeled efficiently with ^99m^Tc and showed high metabolic stability in human plasma in vitro. The data from breast tumor cell binding confirmed the high affinity (in low nanomolar range) towards respective breast cancer cell lines. In healthy mice, ^99m^Tc-labeled peptides displayed favorable pharmacokinetics, with high excretion by the renal system. In tumor xenografts nude mice models, good uptake by the SKBR3, MCF7, and T47D tumors were found, with good tumor-to-blood and tumor to muscle ratios. Additionally, tumor lesions can be seen in γ-camera imaging. Our data suggest that based on its ability to detect HER2- and MUC1-positive breast cancer cells in vivo, ^99m^Tc-HER2 and ^99m^Tc-MUC1-targeted peptides may be promising tumor imaging probes and warrant further investigation.

## 1. Introduction

### 1.1. Tumor Antigens

Tumor antigen is an antigenic substance produced in tumor cells, and causes an immune response in the host. On the basis of the pattern expression, tumor antigens can be classified into two main categories: (i) Tumor-specific antigens (TSA), which are endogenously present only on tumor cells, (ii) tumor-associated antigens (TAA), which are present on both tumor cells and some normal cells, and have been effectively targeted in solid and hematopoietic malignancies. “Overexpressed antigens” are those present at much higher levels in cancer cells than in normal tissues. One of the first tumor-associated antigens described for breast cancer was HER2/neu, a member of the epidermal growth factor receptor family [1,2].

### 1.2. Biology of Tumor-Associated Antigens

The ideal tumor antigen is one that is mainly expressed by malignant cells, thereby providing a distinct target leading to maximal tumor cells killing with low non-target toxicity. While immunotherapy that targets such antigens would be predicted to have minimal side-effects, there is in fact a scarcity of immunogenic tumor-specific antigens. Since most antigens that are expressed by malignant cells are also found in normal tissues, the more accurate designation of these antigens is tumor-associated, rather than tumor-specific. Tumor-associated antigens are distinguished from normal cellular proteins by distinct features in their levels of expression, localization, or major histocompatibility (MHC) processing, allowing for their effective targeting in malignancies [1].

Tumor-associated antigens have been shown to be important targets for immunotherapy for solid and hematopoietic malignancies. The discovery of tumor-associated antigens has allowed for the use of therapies that specifically target malignant cells, thereby sparing normal tissues from the cytotoxic effects of nonspecific chemotherapeutic agents. Some of the noteworthy properties of the ideal tumor-associated antigens include distinct expression by malignant cells as compared to normal cells, proper antigen processing and presentation on cell surface MHC, which together lead to appropriate recognition by the immune system [1,2].

### 1.3. Human Epidermal Growth Factor Receptor 2 (HER2)

Breast cancer is one of the most frequent and the leading type of cancers in women. Early diagnosis and prognosis evaluation will allow for an improved understanding of individual malignancy and proper course of treatment, which ultimately improve the clinical survival of breast cancer. The human epidermal growth factor tyrosine kinase receptor (EGFR/ERBB) family, which includes four members: HER1 (EGFR, ErbB1), HER2 (Neu, ErbB2), HER3 (ErbB3), and HER4 (ErbB4) have been associated with many human cancers. These structurally related proteins coordinate a complex signaling network that plays an important role in the development and evolution of cancer [3,4,5,6].

**Figure 1:**
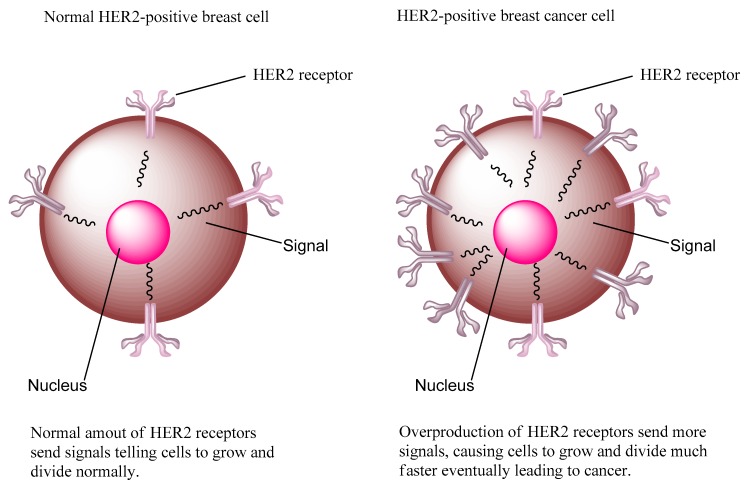
Normal HER2-positive breast cell and HER2-positive breast cancer cell.

HER2 is a 185 kDa transmembrane protein and is a unique receptor molecule due to its lacking of a ligand, but functions as a co-receptor to form homodimers and heterodimers with the other three epidermal growth factor receptor family of tyrosine kinase receptors [5,7,8]. HER2 is overexpressed in many cancers, such as breast, ovarian, endometrial, gastric, pancreatic, and prostate cancers. It has been shown that nearly 75% of all breast cancers express estrogen-receptors (ER) and/or progesterone-receptors (PgR), whereas up to 30% of breast cancers show HER2 overexpression. Around 50% of all HER2-overexpressing breast cancers show the coexistence of both HER2 overexpression/amplification and ER and/or PgR overexpression [7]. Nearly 80–90% of breast cancers express the protein to some extent. Overexpression of the HER2 in breast cancer is classified as “HER2-positive breast cancer” and is considered as one of the most aggressive forms of breast cancer as its status is the marker of poor prognosis, higher mortality in early-stage disease, increased incidence of metastases, and reduced time to relapse comparing with HER2-normal breast cancer (Figure 1). This suggests that HER2 overexpression may be an important initiating event in breast cancer and has become an important validated diagnostic and therapeutic target in breast cancer. [4,5]. Thus, there is a clinical need to explore new and precise methods to visualize HER2 expressing tumors.

Molecular imaging is a promising approach that provides a noninvasive strategy for early tumor diagnosis and real time therapeutic monitoring. The diagnostic imaging of specific cancer-related molecular targets permits earlier and accurate diagnosis of the disease and eventually better management of cancer patients. The two most commonly used molecular imaging modalities for precise diagnosis are single-photon emission computed tomography (SPECT) and positron-emission tomography (PET). SPECT and PET are highly sensitive, noninvasive medical technologies that are suitable for both preclinical and clinical imaging of a wide variety of cancers. Due to the substantial biological and clinical heterogeneity of breast cancer, these sophisticated molecular imaging modalities are capable of measuring tumor receptor expression for the entire tumor burden and provide highly sensitive images of cancer lesion, thus avoiding the sampling error that may occur with heterogeneous tumor receptor expression. Fused PET/CT and SPECT/CT imaging systems now provide metabolic and functional information from PET or SPECT combined with the high spatial resolution and anatomic information of CT. Because the two sets of images are fused, areas of normal and abnormal metabolic activity can be mapped to recognizable anatomic structures. This fusion of function and anatomy has quickly demonstrated its clinical value, especially in the field of nuclear oncology [9]. The recent development of new radiopharmaceuticals now permits molecular imaging of biologic processes at the cellular level to improve both the diagnosis and treatment of disease. For instance, ^68^Ga-DOTATATE and ^68^Ga-PSMA-11 has proved its clinical usefulness in precise diagnosis of a variety of cancers which ultimately resulting in improving therapeutic decisions [10,11].

Molecular imaging probes design for specific targeting of tumor receptors is the important component in molecular imaging. Current biochemical probes for cancer diagnosis mostly rely on the recognition of antibodies toward specific biomarkers on the membrane of cancer cells. However, strong immunogenic response, poor tissue penetration, and slow blood clearance somewhat limit the application of antibodies in cancer. These limitations have stimulated the interest to search for small biomolecule alternatives such as cancer probes/tumor targeting agents. As small molecular probes, peptides own several distinct advantages, such as fast blood clearance, low immunogenicity, good penetration, good biocompatibility, and flexibility for chemical modification. Peptide-based radiopharmaceuticals were introduced into the clinic about three decades ago. The first and most successful FDA-approved neuroendocrine tumors imaging agent to date is the somatostatin analog, ^111^In-DTPA-octreotide (^111^In-OctreoScan). The successful usage of ^111^In-DTPA-octreotide generated enormous clinical interest in the development of radiolabeled peptides to target other tumor-related peptide receptors [10]. A number of peptides identified from peptide libraries are in clinical trials stage and are anticipated to have broad clinical applications with high tumor targeting efficiency while reducing the unwanted side-effects. Tumor-antigens derived peptides are effective targeting probes or vehicles for molecular imaging, which may represent a new horizon for breast cancer diagnostics, staging, and assessments of therapeutic response [3,5,6,10].

### 1.4. HER2-Targeted Antibodies 

As HER2 is overexpressed at the cell surface of tumor cells, the accessibility of the receptor makes it a suitable candidate for molecular imaging and targeted therapy [12]. Several approaches have been used to develop compounds for targeting HER2-positive cancer in the clinical settings. Over the past few years, monoclonal antibodies (mAbs), small peptides, as well as tyrosine kinase inhibitors that target HER2-positive breast cancer have been developed [3,5,13,14,15]. Tyrosine kinase receptors, the monoclonal antibodies (trastuzumab and pertuzumab) have been approved by the FDA (U.S. Food and Drug Administration) for the treatment of HER2 positive breast cancer patients. Recently, HER2 based circulating tumor cells detection is recognized as a valuable prognostic and predictive marker with real-time information to guide individualized therapeutics for breast cancers. However, the above mentioned HER2 recognition was mainly based on antigen−antibody interaction, and small ligands (i.e., peptides) for HER2 are highly desirable [3,12,13,14].

The use of radionuclide molecular imaging would allow for effective detection of HER2 by a noninvasive procedure in both primary tumors and metastases, without false-negative results due to biopsy sampling errors. Clinical utility of radionuclide imaging of HER2 expression using ^111^In-DTPA-trastuzumab made it possible to identify both patients responding to trastuzumab treatment and patients who may suffer from toxicity associated with such treatment. In several preclinical and clinical studies, radiolabeled trastuzumab and pertuzumab have shown high accumulation in tumor tissues. However, the best time for assessment of antibody-based imaging with reasonable tumor-to-organ ratios is typically 3–5 days after administration, which may delay the treatment modification [3,15].

Since the approval of trastuzumab (a humanized monoclonal antibody against the extracellular domain of HER2), three other HER2-targeting agents have obtained regulatory approval. These include: lapatinib (a small molecule tyrosine kinase inhibitor of both HER2 and the epidermal growth factor receptor), pertuzumab (a new anti-HER2 monoclonal antibody), and trastuzumab–emtansine (T-DM1, a novel antibody–drug conjugate), which provide additional treatment options for patients with HER2-positive metastatic breast carcinoma [3,13]. The trastuzumab antibody has improved the outcomes of patients with HER2-positive breast cancer. However, a large number of HER2-positive tumors are not responsive to, or become resistant to, trastuzumab-based therapy, and thus more effective therapies targeting HER2 are needed. [16]. Furthermore, because patients with low and/or heterogeneous HER2 expression have a poorer response to trastuzumab treatment [13], determination of HER2 status is essential in selecting patients for trastuzumab therapy. Molecular imaging is an attractive approach for accomplishing this task; however, using full size monoclonal antibodies such as trastuzumab for imaging is not ideal because of their long retention in blood. Small peptides, on the other hand, are preferred due to their rapid pharmacokinetics and efficient tumor targeting as molecular probes for tumor imaging and targeted therapy [10,17].

### 1.5. HER2-Targeted Peptides 

Phage display is a powerful tool in drug discovery and an effective approach for identifying novel tumor-targeting peptides and their corresponding target receptors. Many such tumor-targeting peptides and their receptors have been successfully used for tumor molecular imaging [18]. Once identified through phage display, selected ligands can be analyzed structurally to provide more detailed understanding of the ligand–target interaction, potency, specificity, and stability. 

The ability to differentiate tumors based on a molecular marker, such as HER2 would allow for detection and categorization of individual breast carcinomas based on the molecular composition of the tumor (personalized precision medicine approach). Although monoclonal antibodies and antibody fragments have high specificity and affinity for their targets, slow clearance, and poor tumor penetration usually hinder their effectiveness as targeting agents. Peptides, in contrast, offer distinctive advantages, such as minimum-sized amino acid sequences with high affinity to their binding sites (targets), no immunogenicity, fast accumulation at the target and rapid blood clearance via the kidneys, site-specific radiolabeling, and cost-effective large-scale production under easy manufacturing-practice conditions. Therefore, HER2-specific peptide ligands have attracted great clinical attention recently. Although numerous peptide-based imaging agents have been synthesized and translated from bench to bed, HER2-targeted peptides for nuclear molecular imaging are found to be inadequate, and thus there is a growing interest to develop HER2-specific peptide for potential clinical application.

Several small peptide molecules have been developed in recent years for targeting HER2-positive breast cancer. For example, a 6-amino-acid containing peptide, KCCYSL, was discovered by the phage display method [19] after in vitro selection with a recombinant ERBB2 extracellular domain. The KCCYSL peptide, derivatized with different metal chelators (i.e., DOTA, NOTA, CB-TE2A, and DAP) and radiolabeled with radionuclide metal ions (e.g., ^111^In, ^64^Cu, ^99m^Tc), has been successfully used for in vivo imaging of HER2 overexpressing tumor models. The tumor cell binding affinity (*K*_d_) of KCCYSL for its target was ranging from 31 to 44 nM. SPECT/CT (Single-positron emission computed tomography/computed tomography) imaging studies showed that radiolabeled peptides were able to accumulate specifically in the HER2-positive tumors [8,19]. Biodistribution studies revealed modest tumor uptake in breast carcinoma xenografts (below 1% ID/g). However, uptake and retention in non-target organs including kidney was high for possible clinical applications. Thus, to make this peptide clinically useful, there is a need to improve the tumor targeting and pharmacokinetic properties of the KCCYSL peptide.

Interestingly, this HER2 peptide sequence has also been proposed for the selective delivery of supramolecular carriers, such as copolymeric micelles or pH-tunable liposomes for diagnostic or therapeutic applications. Bandekar et al. [20] reported a pH-responsive PEGylated DOX liposome modified with KCCYSL. This liposome specifically binds to and internalizes in HER2-positive cells, and subsequently pH-tunable vesicles release DOX rapidly and extensively. In HER2-overexpressing BT474 breast cancer cell-bearing nude mice, this liposome inhibited the tumor growth. These results suggest the potential of pH-tunable vesicles to ultimately control tumor growth at relatively low administered doses [17,20].

A short peptide with the sequence, LTVSPWY, was also identified as a HER2-binding peptide [4]. As an MRI imaging probe, PEGylated chitosan-modified LTVSPWY (LTVSPWY-PEG-CS) was prepared using the solvent-diffusion method. In vitro cytotoxicity assays demonstrated that these magnetic nanoparticles were carriers suitable for use in cancer diagnostics with low toxicity. With proper modification of the LTVSPWY homing peptide, magnetic nanoparticles could be selectively taken up by SKOV-3 cells overexpressing HER2 when co-cultured with HER2-negative A549 cells. In vivo biodistribution data suggest that treatment with LTVSPWY-PEG-CS-modified magnetic nanoparticles/DiR enabled the tumors to be identified and diagnosed more rapidly and efficiently in vivo [21]. Peptide-based nanoprobes may open up new opportunities for the development of novel molecular imaging probes in the early detection and diagnosis of cancer.

An anti-HER2/neu peptidomimetic, (AHNP), FCDGFYACYADVGGG (molecular weight: ~1.5 kDa), was designed from a heavy-chain CDR3 loop of anti-HER2 antibody trastuzumab, and showed functional similarity to Herceptin. AHNP peptide was able to disable HER2 tyrosine kinases in vitro and in vivo similar to the monoclonal antibody from which it was derived. The AHNP mimetic specifically binds to p185HER2/neu with high affinity (*K*_d_ = 150 nM). It has been shown that AHNP diminished the early tyrosine phosphorylation of p185HER2/neu, and inhibited proliferation of p185HER2/neu overexpressing tumor cells in vitro as well as the growth of p185HER2/neu-expressing tumors in athymic mice. Moreover, the mimetic sensitizes the tumor cells to ionizing radiation or chemotherapeutic agents, such as doxorubicin and increases the proportion of cells that undergo apoptosis. These findings suggest that anti-receptor peptide mimetics may prove suitable for clinical use and have some benefits over the full length antibody. The structure-based derivation of AHNP represents an attractive strategy for the design of receptor-specific tumor therapies [22,23,24].

HER2 is usually overexpressed in more than 15% gastric cancer patients so developing a reliable diagnostic tool for tumor HER2 detection is important. Recently, polyethylene glycol (PEG) linked anti-HER2/neu peptide (AHNP-PEG) as a nuclear imaging probe for HER2 detection in gastric cancer xenograft animal model was described [25]. The AHNP-PEG was conjugated with DTPA (diethylenetriaminepentaacetic acid) to facilitate radiolabeling with indium-111 labeling (^111^In-DTPA-AHNP-PEG). These findings suggest that the serum HER2 level measurement may be a potential tool for detecting HER2 expressions in gastric cancer patients. The ^111^In-labeled AHNP-PEG may be useful to apply in gastric cancer patients for HER2 nuclear medicine imaging.

Recently, a new HER2-targeted peptide, H6F (YLFFVFER), was sorted out using a one-bead one-compound combinatorial library approach [15]. The HER2-targeted peptide was conjugated with the bifunctional chelating agent hydrazinonicotinamide (HYNIC) for radiolabeling with ^99m^Tc. The SPECT imaging probe, ^99m^Tc-HYNIC-H6F, exhibited high affinity and high specificity toward HER2 [22]. The ^99m^Tc-HYNIC-H6F peptide probe specifically accumulates in HER2-positive MDA-MBA-453 tumor-bearing mice models using small-animal SPECT/CT imaging and seems promising for the diagnosis of HER2-positive cancers. As ^99m^Tc-HYNIC-H6F and trastuzumab target different regions of the HER2 receptor, this radiopeptide also has good potential for monitoring the therapeutic efficacy of trastuzumab by rechecking the expression level of HER2 without the blocking effect during therapy [15].

In another recent study, a potential HER2-imaging probe, an A9 nonapeptide (Ac-Gln^27^-Asp^28^-Val^29^-Asn^30^-Thr^31^-Ala^32^-Val^33^-Ala^34^-Trp^35^-NH_2_), derived from the trastuzumab-Fab portion which is capable to bind HER2 was evaluated [26]. ^111^In-DTPA-labeled A9 peptide demonstrated nanomolar affinity to HER2-expressing BT474 cells in vitro and favorable in vivo biodistribution properties that can be characterized by rapid clearance from the blood and low uptake by the major organs and tissues in NMRI mice. This study suggests that the peptide A9 represents a good lead candidate for development of HER2 molecular probe to be used for molecular imaging purposes and for the delivery of cytotoxic agents.

We recently constructed a hybrid HER2-targeting peptide probe by linking two important HER2-derived peptide sequences [27]. For this purpose, the hexapeptide, KCCYSL, which bound the extracellular domain of human HER2 and has shown the potential for targeting HER2 positive human breast and prostate cancers was selected [19]. Another HER2 targeted peptide that was chosen based on E75 (369‒377) sequence of HER2 protein had shown to be overexpressed in many breast cancer patients. The E75 (KIFGSLAFL; HER2, 369‒377) sequence derived from HER2 protein’s extracellular region has also been employed in the formulation of peptide-based cancer vaccine (nelipepimut-S) to prevent breast cancer recurrence in high-risk patients [28,29]. It seems reasonable to presume that this sequence can be useful to formulate a HER2-based peptide for targeting HER2-positive breast cancer. In an attempt to enhance targeting efficiency of HER2-targing vector, we have combined the HER2 binding amino acids sequences of these two important peptides to construct a HER2-targeted hybrid peptide as an SPECT imaging probe in order to evaluate its tumor targeting potential in vivo in subcutaneous HER2-positive human breast cancer xenografts models. The HER2 hybrid peptide (Acetyl-Gly^19^-Gly^18^-Cys^17^-ALA^16^-Lys^15^-Ile^14^-Phe^13^-Gly^12^-Ser^11^-Leu^10^-Ala^9^-Phe^8^-Leu^7^-Lys^6^-Cys^5^-Cys^4^-Tyr^3^-Ser^2^-Leu^1^-CONH_2_ was prepared by Fmoc-based solid-phase peptide synthesis and radiolabeled with technetium-99m (^99m^Tc) via Gly-Gly-Cys- chelating sequence. ^99m^Tc is the radionuclide of choice in the development of tumor targeting peptides owing to its wide availability, suitable half-life (6 h), and ideal γ-energy (140 keV) for medical diagnostic imaging [27]. 

### 1.6. Mucin 1 (MUC1)

Differentially regulated proteins on the surface of cancer cells, such as the tumor-specific antigens, are potential molecular targets for the development of alternative and effective anticancer agents. One such target is localized on the apical region of normal epithelial cells, but gets aberrantly overexpressed in various cancers in human epithelial mucin 1 (MUC1), which received considerable interest as a cancer antigen target [30,31]. MUC1 was chosen as a model tumor antigen as it is the best characterized human tumor cell-surface antigen, which has been developed as a tumor marker for clinical use, in particular as a marker of human breast cancer [32]. MUC1 is overexpressed on the entire cell surface in a wide variety of cancers (i.e., lung, prostate, gastrointestinal tract, etc.), especially by primary and metastatic breast cancers [33]. MUC1 overexpression enhances tumor invasiveness and aggressiveness and promotes metastasis in breast cancers [34,35]. Due to the overexpression of MUC1, which correlates with high metastatic potential and poor patient survival, the ability to target such tumors may be highly beneficial in clinical settings for both imaging and treatment of a variety of tumors. It should be noted that MUC1 overexpression is not limited to breast cancer alone as it is highly expressed on the entire cell surface of a wide range of carcinomas including prostate, lung, gastrointestinal tract, and other epithelia [36]. 

**Figure 2:**
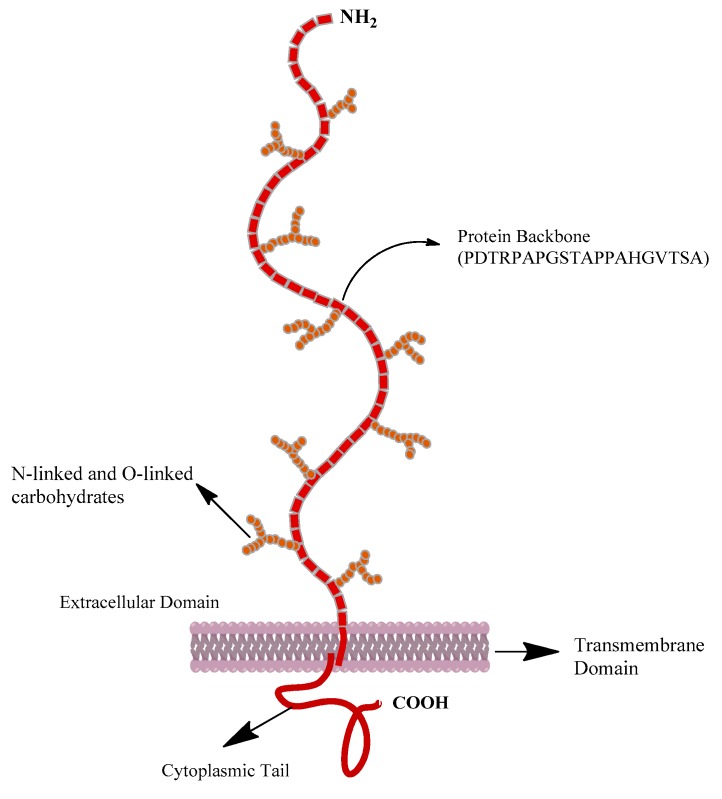
Structure of mucin 1 (MUC 1) [38].

MUC1 is a breast cancer-associated type I transmembrane heterodimer, comprised of two subunits: An α-subunit consisting of an extracellular N-terminal domain and a β-subunit composed of a transmembrane helix and a short cytoplasmic tail (Figure 2) [37,38]. The extracellular domain of MUC1 contains a variable number of tandem repeats consisting of 20–120 repeats of a 20-amino acid sequence N-**PDTRP**APGSTAPPAHGVTSA-C [39,40]. The distinctive extracellular domain of MUC1 is defined by the presence of the amino acid sequence PDTRP, which is the minimal MUC1 core peptide sequence (shown in bold above). The same sequence is also recognized by several highly tumor-specific anti-mucin monoclonal antibodies [41,42,43], as well as some patients’ sera [44]. Therefore, we hypothesized that this sequence could be useful to design a synthetic MUC1 peptide derived from the tumor-antigen for targeting MUC1-positive breast carcinoma. It is expected that the overexpression of MUC1 on breast cancer would facilitate target specific imaging and therapy using synthetic MUC1-derived peptide. We have synthesized, by solid-phase synthesis, a novel MUC1-derived peptide (Ac-GGCEPDTRP-amide) based on PDTRP sequence and coupled to Gly-Gly-Cys (GGC, triamide-thiol) chelating sequence to facilitate radiolabeling with technetium-99m (^99m^Tc). A negatively-charged glutamic acid residue was inserted as a spacer group between the MUC1 binding region and the chelating sequence [45]. The GGC-based chelating sequence is used because of its ease of synthesis and direct coupling to the tumor targeting peptide as well as its well-defined labeling chemistry. Preclinical evaluation of the newly developed HER2 hybrid peptide and MUC1 peptide for the detection of breast cancer is briefly presented here. 

## 2. Results and Discussion

### 2.1. Peptide Synthesis and Radiolabeling with ^99m^Tc 

**Figure 3:**
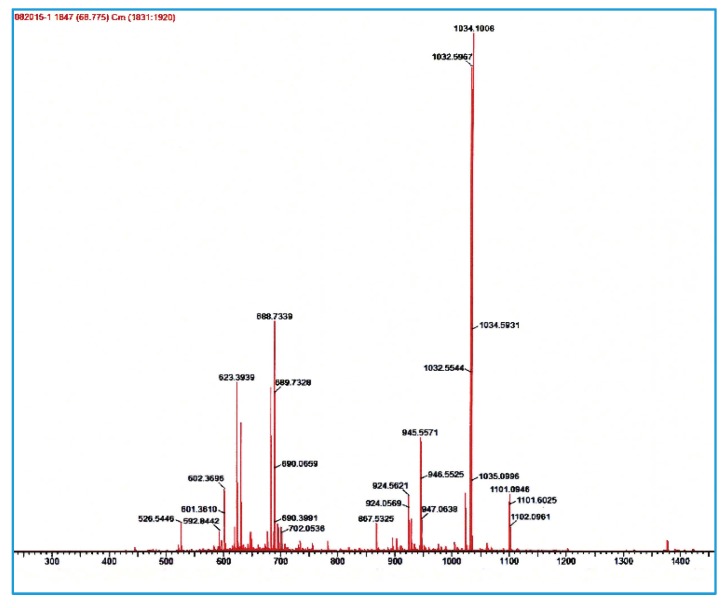

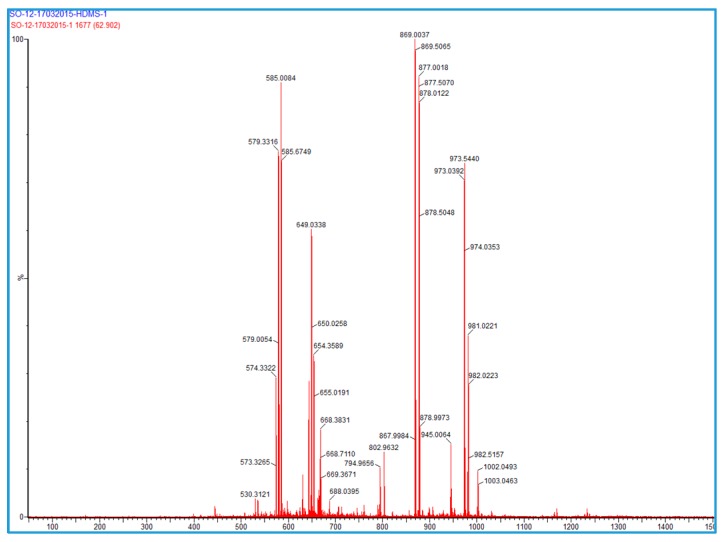
Mass spectrum of HER2 peptide (above) and MUC1 peptide (below). ESI-MS: HER2: [M + H]^+^ calculated = 2064; [M + 2H]^2+^ found = 1034; For MUC1: [M + H]^+^ calculated = 972; found = 973.

Both HER2 and MUC1 peptide investigated in this study were conveniently and successfully prepared by solid-phase peptide synthesis according to standard Fmoc-chemistry in satisfactory yield (~40%). The purity of each peptide was confirmed by HPLC analysis and structural authenticity by mass spectrometry. The ESI-MS for MUC1: [M + H]^+^ calculated = 972, found = 973; and for HER2: ([M + H]^+^ calculated = 2064; [M + 2H]^2+^ found = 1034) (see Figure 3). 

**Figure 4:**
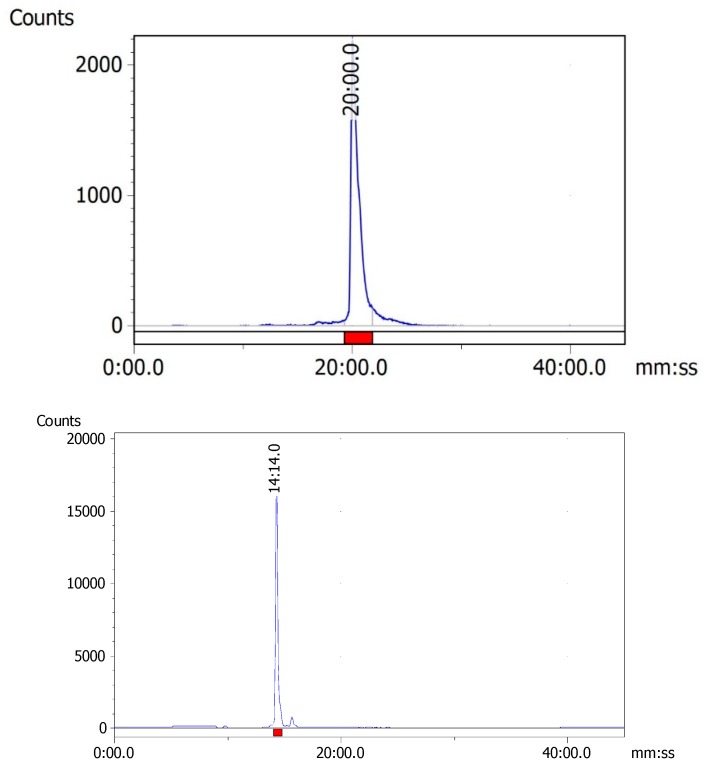
Radio-HPLC chromatograms of ^99m^Tc-HER2 (above) and ^99m^Tc-MUC1 (below).

The HER2 and MUC1 synthetic peptides were radiolabeled efficiently with ^99m^Tc by the standard ligand exchange method using sodium potassium tartrate as a weak chelating agent. By the exchange labeling approach, the radiolabeling efficiency of both ^99m^Tc-HER2 and ^99m^Tc-MUC1 was found to be greater than 95%. Radio-HPLC analysis revealed the formation of one major peak corresponding to ^99m^Tc-HER2 at 20.0 min, whereas the peak for ^99m^Tc-MUC1 was found at 14.1 min (Figure 4). The radiochemical purity was determined by evaluating radioactivity peak eluted for each ^99m^Tc-labeled peptide from the RP-HPLC column and calculating the counts under the peak (region of interest). The relatively longer retention time of ^99m^Tc-HER2 complex on HPLC is possible due to its higher lipophilicity than ^99m^Tc-MUC1. Some characteristics of the synthesized peptides are presented in Table 1. 

### 2.2. In Vitro Tumor Cell Binding 

^99m^Tc-HER2 displayed a high binding affinity toward HER2-positive SKBR3 breast cancer cell line, with the *K*_d_ value of 49.97 ± 14.15 nM. However, for ER-positive breast cancer cell lines (i.e., MCF7 and T47D), ^99m^Tc-HER2 exhibited a significantly lower binding affinity, with *K*_d_ values of 157.92 ± 24.60 nM and 169.44 ± 22.29 nM, respectively. These findings suggest that the binding affinity of ^99m^Tc-HER2 was found to be more than 3-fold higher for HER2 overexpressing SKBR3 cells than the low expressing HER2-positive and ER-positive MCF7 and T47D cell lines. A fast and significant internalization was observed for ^99m^Tc-HER2 since 19.90 ± 5.35% of the cell-surface bound radiopeptide was internalized into SKBR3 cells after incubation in acidic buffer for 10 min at 37 °C. The internalization capability of two ER-positive cell lines was found to be relatively lower than HER2-positive SKBR3 cell line as only 12.16 ± 3.20% and 11.85 ± 2.83% of the cell bound radioactivity was internalized into MCF7 and T47D cells, respectively. The results of cell-binding and internalization into various breast cancer cells demonstrate that, in spite of the modifications in the peptide sequence, such as the introduction of a chelating sequence for radiolabeling and the addition of spacer group, the HER2-derived peptide retained its potency and held sufficient affinity and specificity for breast cancer cell lines. This highlights the potential of ^99m^Tc-HER2 for targeting human breast cancer (Table 2).

^99m^Tc-MUC1-derived peptide was also evaluated for its capacity to bind with two well-characterized human breast cancer cell lines, such as the human ductal breast cancer cell line T47D and estrogen-receptor positive MCF7. These MUC1-positive cell lines present a diverse range of receptor expression [46,47]. ^99m^Tc-MUC1 showed 3-fold higher binding affinity to T47D cell line (*K*_d_ = 3.48 ± 0.84 nM), as compared to MCF7 cell line (*K*_d_ = 10.30 ± 1.55 nM). A rapid and significant internalization was observed for ^99m^Tc-MUC1 with, 21.11 ± 2.95% and 18.92 ± 2.16% of the cell-surface bound radiopeptide internalized into T47D and MCF7 cells, respectively, after incubation in acidic buffer for 10 min at 37 °C. The results of cell-binding and internalization into tumor cells demonstrate that the ^99m^Tc-MUC1 peptide maintained its potency and held high affinity and specificity towards receptor-positive breast carcinomas. This underlines the potential of ^99m^Tc-MUC1 peptide for targeting human breast cancers (Table 2).

### 2.3. In Vivo Biodistribution and Tumor Uptake Studies of ^99m^Tc-HER2

**Figure 5:**
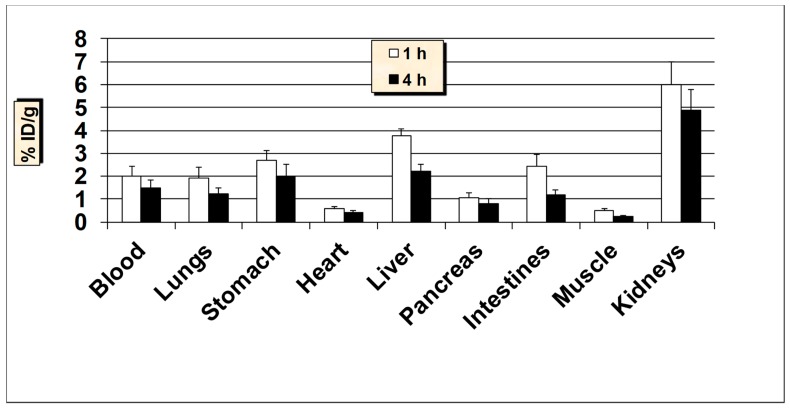
In vivo tissue biodistribution of ^99m^Tc-HER2-derived peptide in normal Balb/c mice at 1 h and 4 h post-injection. Data is represented as % injected dose per gram (% ID/g) of tissue/organ (n = 3–5, mean values ± SD). Part of the intestines was measured without their contents.

All animal studies were conducted according to the international regulations governing the safe and proper use of laboratory animals [48]. Approval for the animal protocol used in these studies was obtained from the Institutional Animal Care and Use Committee. Initial biodistribution studies of ^99m^Tc-HER2-derived peptide was performed in normal Balb/c mice at 1 and 4-h post-injection (p.i.) and the results are summarized in Figure 5. The results demonstrate that the ^99m^Tc-HER2 displayed a fast and efficient clearance from the blood as less than 2% ID/g was remained in the blood at 1 and 4 h p.i. The initial uptake by the liver was 3.74 ± 0.71% ID/g at 1 h which reduced to 2.18 ± 0.38% ID/g at 4 h p.i. The reasonable uptake of ^99m^Tc-HER2 peptide in the liver is probably due to its modest lipophilicity (log P = 1.38 ± 0.06). The intestinal uptake (without the contents) was also low (up to 2.41 ± 0.42% ID/g) at both 1 and 4 h p.i. While the observed kidney uptake (5.96 ± 1.03% ID/g at 1 h and, 4.85 ± 0.86% ID/g at 4 h p.i.) is not very high, it still has a low uptake and retention by the kidneys desired for diagnostic imaging in general and for radionuclide therapy in particular, because of possible kidney toxicity [49]. A low to moderate uptake of radioactivity was observed in the stomach (range, 2.66–2.0% ID/g), indicating a low breakdown of the ^99m^Tc-HER2 complex in vivo and minimal reformation of free ^99m^TcO_4_^−^. Nonetheless, the high in vivo stability for ^99m^Tc-HER2 peptide correlates well with the high in vitro metabolic stability obtained in plasma. The uptake in the lungs was also low (below 2% ID/g both at 1 and 4 h p.i.) indicating a low trapping by lungs. The data suggest that the moderately lipophilic radiopeptide (log P = 1.38 ± 0.06), with ALA (aminoleuvinic acid) as a spacer group displayed good urinary excretion property as up to 40.0 ± 5.10% ID was found in the urine. The clearance pathway highlights the significance of the extent of lipophilicity as one of the determining factors in the clearance passage of a peptide radiopharmaceutical. In general, a rapid and efficient clearance of the radioactivity was observed from most of the major organs underlining the overall favorable biodistribution behavior of the ^99m^Tc-HER2-derived peptide.

**Ethics approval:** Approval for the animal protocol used in this study was obtained from the Institutional Animal Care and Use Committee (RAC number: 2050042; King Faisal Specialist Hospital and Research Centre, Riyadh, KSA-ACUC).

**Figure 6:**
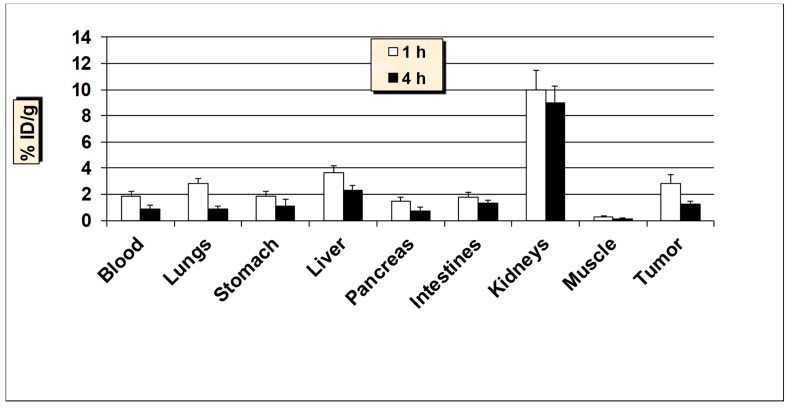
In vivo tumor targeting studies of ^99m^Tc-HER2-derived peptide in HER2-positive SKBR3 breast cancer xenografts mice models. Data is represented as % injected dose per gram (% ID/g) of tissue/organ (n = 3–5, mean values ± SD). Part of the intestines was measured without their contents.

Encouraged by the favorable pharmacokinetics in normal mice, ^99m^Tc-HER2 peptide was further investigated in nude mice bearing HER2-positive SKBR3 tumor xenografts in order to determine its capability to target human breast cancer in vivo (Figure 6). Additionally, in tumor-bearing nude mice, a rapid and effective clearance from the blood was observed similar to Balb/c mice. A good uptake of 2.81 ± 0.79% ID/g was observed in the HER2-positive SKBR3 tumors as early as 1 h p.i., which reduced to 1.22 ± 0.25% ID/g at 4 h p.i. (with 57% washout from the tumors over 4 h). The uptake value in the tumor was always higher than the radioactivity found in the blood and muscle. A tendency of slight decreased tumor-to-blood and tumor-to-muscle uptake ratios over time was obtained for ^99m^Tc-HER2 peptide. The tumor-to-blood ratio obtained was 1.54 at 1 h, which dropped to 1.34 at 4 h p.i., whereas tumor-to-muscle ratio was found to be 9.37 at 1 h, which reduced to 8.71 at 4 h p.i. Uptake in the stomach was found to be lower in nude mice than that the Balb/c mice. The kidneys showed the highest accumulation and retention of radioactivity (up to 10% ID/g) and these values are about 2-times higher than the values found in the Balb/c mice. A high amount of radioactivity was excreted in the urine (up to 41% ID) whereas, the hepatobiliary excretion (liver + intestines) was below 15% ID. In a receptor blocking study (where 100 μg of HER2 peptide was administered 30 min before the injection of ^99m^Tc-HER2), uptake in the tumors was reduced by approximately 62% (1.07 ± 0.24% ID/g blocked vs. 2.81 ± 0.79% ID/g unblocked, *P* = 0.02), highlighting the specificity of the ^99m^Tc-HER2-derived peptide for the respective HER2-positive SKBR3 breast cancer cell line. A good and specific uptake by the tumors combined with decent tumor to background uptake ratios advocates the possible potential of this tumor-antigen derived peptide for targeting breast cancer.

**Figure 7:**
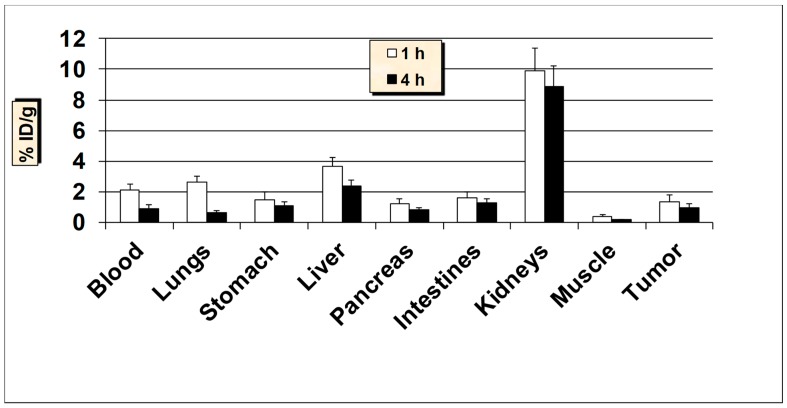
In vivo tumor-targeting properties of ^99m^Tc-HER2 peptide in ER-positive MCF7 breast cancer xenografts mice models. Data is represented as % injected dose per gram (% ID/g) of tissue/organ (n = 3–5, mean values ± SD). Part of the intestines was measured without their contents.

**Figure 8:**
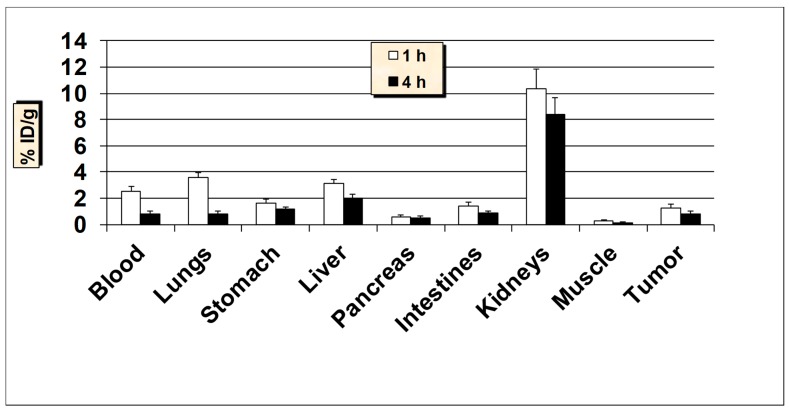
In vivo tumor-targeting properties of ^99m^Tc-HER2 peptide in ER-positive T47D breast cancer xenografts mice models. Data is represented as % injected dose per gram (% ID/g) of tissue/organ (n = 3–5, mean values ± SD). Part of the intestines was measured without their contents.

The tumor targeting ability of ^99m^Tc-HER2-derived peptide was also investigated in nude mice with estrogen receptor-positive MCF7 and T47D tumor xenografts (Figure 7 and Figure 8). The estrogen receptor-positive cell lines, MCF7 and T47D are considered as low HER2 expressing breast cancer cell lines [50,51]. A fairly comparable tumor uptake and tissue biodistribution profiles were obtained between the nude mice models carrying estrogen receptor-positive MCF7 and T47D breast cancer xenografts. The uptake of ^99m^Tc-HER2 in the MCF7 and T47D tumors was 1.33 ± 0.41% ID/g and 1.25 ± 0.32 at 1 h and 0.91 ± 0.22% ID/g and 0.80 ± 0.17% ID/g at 4 h p.i., respectively. These results demonstrate that the uptake by estrogen receptor-positive tumors was about 2-fold lower than the uptake found in HER2-positive SKBR3 breast carcinoma. The data highlight the strength of ^99m^Tc-HER2-derived peptide to target not only HER2-positive, but also estrogen receptor-positive breast cancer lines.

**Figure 9:**
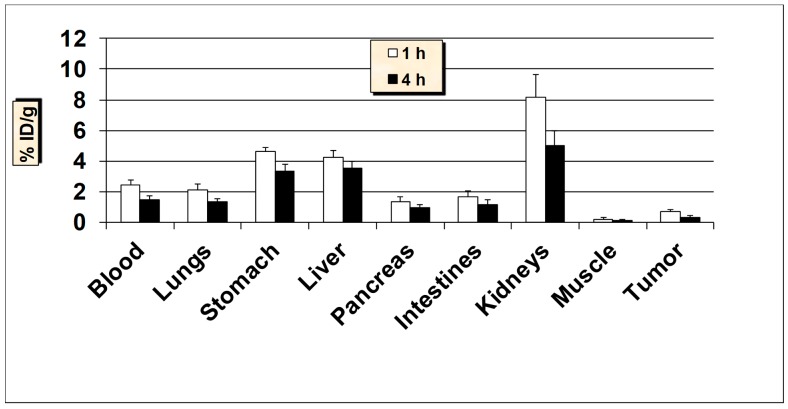
In vivo tumor-targeting properties of ^99m^Tc-HER2 peptide in HER2-negative MDA-MB-231 breast cancer xenografts mice models. Data is represented as % injected dose per gram (% ID/g) of tissue/organ (n = 3–5, mean values ± SD). Part of the intestines was measured without their contents.

In addition, tumor-targeting behavior of ^99m^Tc-HER2 peptide was evaluated in nude mice bearing HER2-negative MDA-MB-231 breast xenografts to further examine the tumor specificity of the radiopeptide by comparing the extent of tumor uptake in HER2-positive and HER2-negative cancer cell lines (Figure 9). MDA-MB-231 is classified as HER2-negative breast cancer cell line because of its low-expression for HER2 [15,52]. The tumor uptake value of 0.66 ± 0.13% ID/g vs. 2.81 ± 0.79% ID/g (*P* = 0.009) at 1 h, and 0.31 ± 0.10% ID/g vs. 1.22 ± 0.25% ID/g (*P* = 0.004) at 4 h was found in HER2-negative MDA-MB-231 versus HER2-positive SKBR3 tumors, respectively. These results validate the significant tumor uptake specificity by ^99m^Tc-HER2 peptide for HER2-positive SKBR3 cell line over HER2-negative MDA-MB-231 tumor xenografts. No noticeable difference in uptake patterns by the major organs was seen between the HER2-positive and HER2-negative tumor xenograft models.

**Figure 10:**
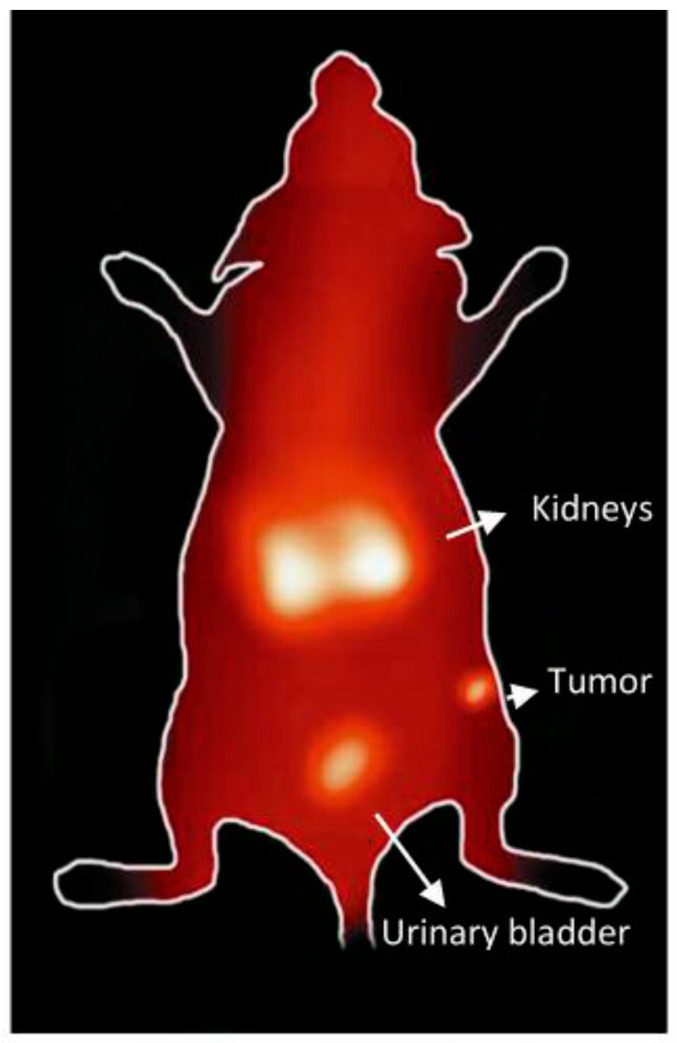
Gamma camera full body image of nude mouse implanted with HER2-positive. SKBR3 xenografts at 1 h post-injection of ^99m^Tc-HER2 peptide.

The tumor imaging property of ^99m^Tc-HER2-derived peptide was examined by gamma camera imaging of a nude mouse bearing HER2-positive SKBR3 tumor xenografts at 1 h after the administration of radiopeptide. Although a high accumulation of the radioactivity in the kidneys and urinary bladder is seen, the SKBR3 xenografted tumor is detectable in the image at 1 h p.i. (Figure 10), highlighting the potential use of tumor-antigen derived HER2 peptide for human breast cancer imaging. It was observed that among the normal organs, kidney displayed the highest radioactivity uptake in the biodistribution and gamma image studies. Therefore, in order to make ^99m^Tc-HER2-derived peptide more suitable for tumor imaging, besides the strategy to decrease kidney uptake, it is also important to increase the tumor uptake in future studies. This can be done by carrying the proper modification in the peptide sequence or using the suitable peptide cocktail in order to enhance tumor targeting efficiency and pharmacokinetics.

### 2.4. In Vivo Biodistribution and Tumor Uptake Studies of ^99m^Tc-MUC1

**Figure 11:**
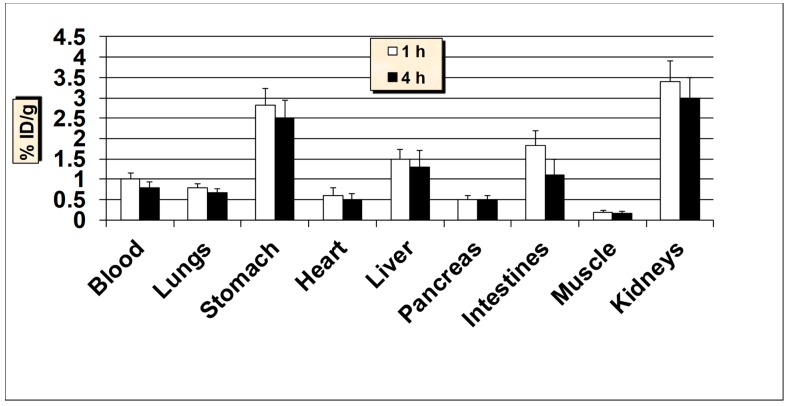
In vivo tissue biodistribution studies of ^99m^Tc-MUC1-derived peptide in normal Balb/c mice at 1 h and 4 h post-injection. Data is presented as % injected dose per gram (% ID/g) of tissue/organ (n = 3–5, mean values ± SD). Parts of the intestines were measured without their contents.

The results of biodistribution studies in normal Balb/c mice at 1 h and 4 h post-injection (p.i.) of ^99m^Tc-MUC1-derived peptide are graphically presented in Figure 11. The biodistribution results from healthy mice suggest that the ^99m^Tc-MUC1-derived peptide revealed a rapid and efficient clearance from the blood both at 1 h and 4 h p.i., with less than 1% ID/g remained in the blood after 4 h p.i. The radioactivity accumulated by the liver was 1.50 ± 0.13% ID/g at 1 h and 1.28 ± 0.16% ID/g at 4 h p.i. possibly due to its high hydrophilicity. The uptake in the intestines (without the contents) was also low (up to 1.81 ± 0.32% ID/g) at both 1 h and 4 h p.i., but when radioactivity was measured in the intestines with contents, a much higher accumulation and retention (up to 7.51 ± 1.23% ID/g) was observed. The ^99m^Tc-MUC1-derived peptide exhibited a moderate accumulation and retention in the kidneys (3.40 ± 0.42% ID/g at 1 h and, 2.95 ± 0.40% ID/g at 4 h p.i.). Low kidney uptake and retention is desirable for medical diagnostic imaging in general and for radionuclide therapy in particular, because of potential nephrotoxicity [49]. A low to moderate uptake of radioactivity was observed in the stomach (range, 2.49–2.83% ID/g), indicating a minimal reformation of free ^99m^TcO_4_^−^. Nevertheless, the high in vivo stability for ^99m^Tc-MUC1 peptide correlates well with the high in vitro metabolic stability obtained in plasma. The uptake in the lungs was also low (less than 0.80% ID/g both at 1 h and 4 h p.i.) indicating low colloidal particles formation. The data suggest that the introduction of a negatively-charged and hydrophilic spacer (glutamic acid) in the peptide sequence significantly enhanced the renal excretion of the radiopeptide as up to 47.0 ± 6.28% ID was found in the urine in accordance with its high hydrophilic property (log P = −2.22 ± 0.12). The clearance pattern underlines the importance of the degree of lipophilicity as one of the determining factors in the clearance passage of a peptide-based radiopharmaceutical. In general, a rapid and efficient clearance of the radioactivity was observed from all the major organs, highlighting the overall favorable biodistribution profile of the ^99m^Tc-MUC1-derived peptide.

**Figure 12:**
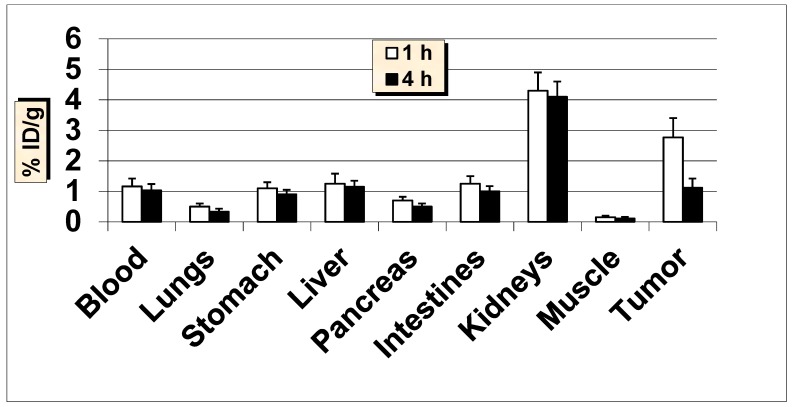
In vivo tumor targeting of ^99m^Tc-MUC1-derived peptide in MUC1-positive MCF7 tumor bearing female nude mice at 1 and 4 h post-injection. Data are expressed as % injected dose per gram (% ID/g) of tissue/organ (n = 3, mean values ± SD). Parts of the intestines were measured without their contents.

**Figure 13:**
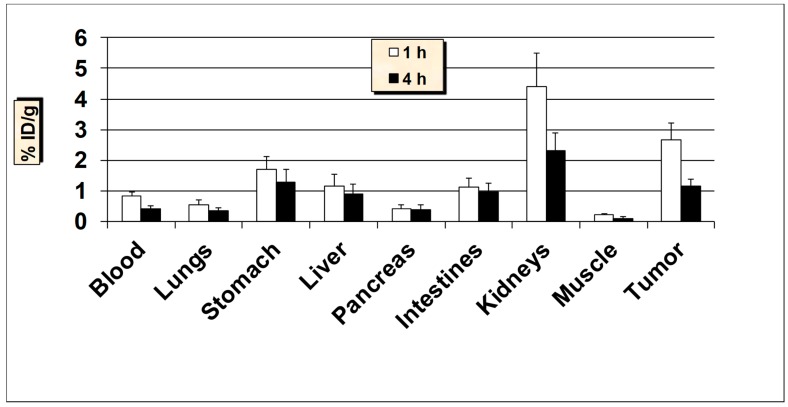
In vivo tumor targeting of ^99m^Tc-MUC1-derived peptide in MUC1-positive T47D tumor bearing female nude mice at 1 and 4 h post-injection. Data are expressed as % injected dose per gram (% ID/g) of tissue/organ (n = 3, mean values ± SD). Parts of the intestines were measured without their contents.

In order to determine its ability to target human breast cancer in vivo, ^99m^Tc-MUC1-derived peptide was further tested in nude mice bearing MUC1-positive MCF7 and T47D xenografts (Figure 12 and Figure 13). Additionally, in nude mice, a rapid clearance from the blood was obtained, as was the case with Balb/c mice. A good uptake of 2.77 ± 0.63% ID/g was found in the tumors as early as 1 h p.i., which reduced to 1.12 ± 0.30% ID/g at 4 h p.i. (with 60% washout from the tumors over 4 h). The uptake value in the tumor was always higher than the radioactivity in the blood and muscle. A trend of decreased tumor-to blood and tumor-to-muscle uptake ratios over time was obtained for ^99m^Tc-MUC1 peptide. The tumor-to-blood ratio obtained was 2.39 at 1 h, which reduced to 1.09 at 4 h p.i., whereas tumor-to-muscle ratio was found to be 18.47 at 1 h, which somewhat dropped to 11.20 at 4 h p.i. Uptake of radioactivity in the lungs and stomach was found to be lower in nude mice than that the Balb/c mice. The accumulation in the kidneys was moderate but somewhat higher than the value found in the Balb/c mice. The radioactivity excreted in the urine was up to 49% ID, whereas the hepatobiliary excretion (liver + intestines) of this radiopeptide was below 13% ID. A fairly high uptake by the tumors combined with good tumor to background uptake ratios advocates the possible potential of this tumor-specific antigen peptide for targeting human breast cancer.

In nude mice with T47D tumor xenografts, ^99m^Tc-MUC1-derived peptide exhibited fast and efficient clearance from the blood, with 0.30 ± 0.07% ID/g remaining in the blood circulation at 4 h p.i. (Figure 13). The uptake in the tumor was found to be 2.65 ± 0.54% ID/g at 1 h and 1.14 ± 0.21% ID/g at 4 h p.i., respectively. The tumor-to-blood and tumor-to-muscle ratios were found to be 3.27 and 12.62 at 1 h and 2.85 and 11.40 at 4 h p.i., respectively. A somewhat comparable tumor uptake and tissue biodistribution profile is obtained between the nude mice models carrying estrogen-receptor positive MCF breast cancer cell line and T47D human ductal breast epithelial cancer cell line xenografts. The similar in vivo tumor targeting behavior of ^99m^Tc-MUC1 peptide in both T47D and MCF7 tumor xenografts is rather contrary to the in vitro binding characteristics, where ^99m^Tc-MUC1 peptide exhibited nearly 3-fold higher affinity for T47D human ductal breast cancer cell line than MCF7 cell line. Initial accumulation of the radiopeptide in the kidneys was 4.35 ± 1.0 but reduced to 2.26 ± 0.36 at 4 h p.i. (48% cleared over 4 h), signifying the low retention by the kidneys resulting in efficient clearance of the radiopeptide through the kidneys into the urine. ^99m^Tc-MUC1 peptide showed high levels of the excretory radioactivity in the urine, 50.0 ± 9.14% ID and 48.0 ± 8.23% ID at 1 h and 4 h p.i., respectively, suggesting that the major route of excretion was the renal system. The hepatobiliary excretion was below 13% ID at both 1 h and 4 h p.i. It is apparent from the biological evaluation that the ^99m^Tc-MUC1 peptide displayed comparable tumor targeting characteristics in the species carrying two pharmacologically distinct human breast cancer cell lines (MCF7 and T47D), highlighting the ability of ^99m^Tc-MUC1 peptide to target various MUC1 positive breast cancer lines.

Receptor blocking study was also carried out where 100 μg of unlabeled MUC1 peptide was administered 30 min before the injection of ^99m^Tc-MUC1 peptide. It was found that the blocking dose reduced the uptake in the tumors by approximately 62% (1.05 ± 0.21% ID/g blocked vs. 2.77 ± 0.63% ID/g unblocked, *P* = 0.02), underlining the specificity of the ^99m^Tc-MUC1-derived peptide for respective MUC1-positive breast cancer cell line. No marked influence of the blocking dose was observed in other major organs and tissues.

**Figure 14:**
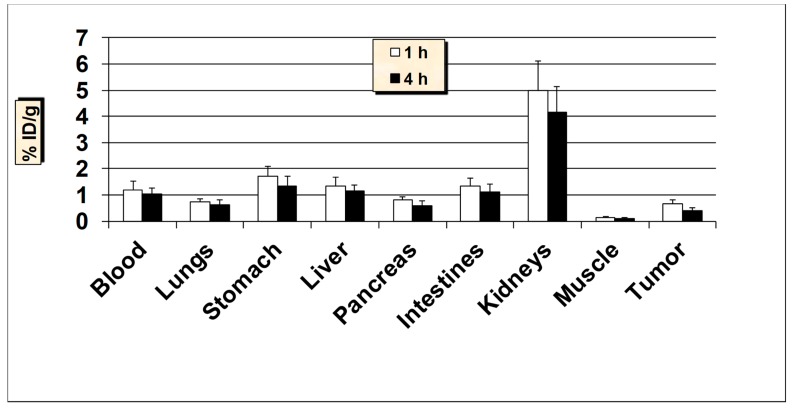
In vivo tumor targeting of ^99m^Tc-MUC1-derived peptide in MUC1-negative MDA-MB-231 tumor bearing female nude mice at 1 and 4 h post-injection. Data are expressed as % injected dose per gram (% ID/g) of tissue/organ (n = 3, mean values ± SD). Parts of the intestines were measured without their contents.

Additionally, tumor targeting evaluation was carried out in nude mice bearing MUC1-negative MDA-MB-231 breast cancer line (Figure 14) in order to further examine the tumor specificity of ^99m^Tc-MUC1 peptide by comparing the extent of tumor uptake in MUC1-positive and MUC1-negative tumor xenograft models. MDA-MB-231 is considered as MUC1-negative breast cancer cell line due to the low expression capacity for MUC1 [47,53]. A tumor uptake of 0.64 ± 0.13% ID/g vs. 2.77 ± 0.63% ID/g (*P* = 0.009) at 1 h, and 0.41 ± 0.10% ID/g vs. 1.12 ± 0.30% ID/g (*P* = 0.01) at 4 h was found in MUC1-negative (MDA-MB-231) versus MUC1-positive (MCF7) tumor, respectively, advocating the significant tumor uptake specificity by ^99m^Tc-MUC1 peptide for MUC1-positive breast cancer cell line over MUC1-negative MDA-MB-231 xenografts. No marked difference in uptake patterns by the major organs was seen between the MUC1-positive and MUC1-negative tumor xenograft models. Taken together, the favorable biodistribution, combined with good specificity in vivo tumor targeting characteristics rendering ^99m^Tc-MUC1 peptide as a potential candidate for targeting MUC1-positive tumors.

**Figure 15:**
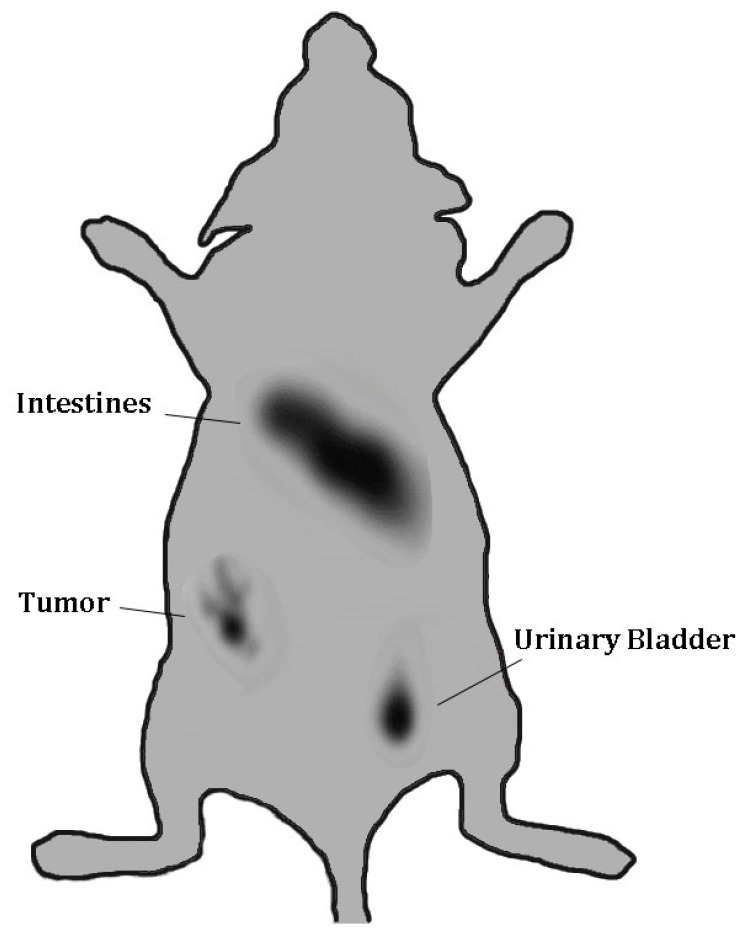
Gamma camera full body image of nude mouse implanted with MUC1-expressing. MCF7 tumor xenografts at 1 h post-injection of ^99m^Tc-MUC1 peptide.

A nude mouse with MUC1-positive MCF7 tumor xenograft was imaged by gamma camera at 1 h after the administration of ^99m^Tc-MUC1 peptide. While a high accumulation of the radioactivity in the abdominal region and urinary bladder is seen, the tumor lesion is detectable in the image at 1 h p.i. (Figure 15), underlining the tumor targeting ability of the tumor-antigen derived peptide. From the biodistribution data, it seems that ^99m^Tc-MUC1 peptide shows good tumor targeting properties and favorable overall biodistribution profile and appears to be a potential candidate for breast cancer targeting and deserves further evaluation.

## 3. Materials and Methods

### 3.1. Solid-Phase Synthesis of HER2 and MUC1 Peptides

The HER2 and MUC1-derived peptides were synthesized manually in a silanized 15 mL glass reaction vessel by solid-phase peptide synthesis following standard Fmoc (9-fluorenylmethoxycarbonyl) chemistry, using Rink amide MBHA (4-methylbenzhydrylamine) resin (100–200 mesh) on a 0.2 mmol scale according to a general method of peptide synthesis described previously [54]. Briefly, the first Fmoc-amino acid of each peptide sequence was activated in situ with HBTU (*O-*benzotriazol-1-yl)-1,1,3,3-tetramethyluronium hexafluorophosphate) in the presence of DIEA (diisopropylethylamine) and mixed with the peptide resin. Each peptide chain was elongated in cycles of Fmoc-deprotection followed by the coupling of the subsequent Fmoc-amino acid to the resin. After incorporating all the desired amino acids to the peptide resin, the N-terminal Fmoc-protecting group was removed with 20% piperidine/DMF solution and the peptide-resin was acetylated with acetic anhydride in the presence of triethylamine. Cleavage of the crude peptides and concomitant removal of the side-chain protecting groups was accomplished with a mixture of 94% TFA (trifluoroacetic acid), 1% triisopropylsilane, 2.5% 1,2-ethanedithiol, and 2.5% water. The purity of each peptide was confirmed by HPLC (Shimadzu Corporation, Kyoto, Japan) and their structural identities by mass spectrometry.

### 3.2. Radiolabeling with ^99m^Tc

A 25–50 μL of each peptide solution (1 mg/mL CH_3_CN/H_2_O) was mixed with 200 μL 0.2 M citrate–phosphate buffer (pH 9), 250 μL sodium potassium tartrate (40 mg/mL aqueous solution) and 20 μL 5% ascorbic acid. To this, freshly prepared 100 μL of SnCl_2_·2H_2_O in 0.05 N HCl (25 mg of SnCl_2_·2H_2_O in 5 mL of 0.05 N HCl) was added followed by 200 μL of ^99m^TcO_4_^−^ (5–10 mCi) (Poltechnet ^99^Mo/^99m^Tc generator, Polatom, Poland). The labeling mixture was then heated at 90 °C for 10 min and allowed to cool to room temperature prior to HPLC analysis. The preparation was filtered through 0.22 μm pore syringe filter to remove any precipitate.

### 3.3. HPLC Purification and Analysis

The HPLC analysis and purification of the peptides were performed on a Shimadzu HPLC system using Econosphere C18 reversed-phase column (10 μm, 250 × 4.6 mm, Alltech, Deerfield, IL, USA). For HPLC experiments, a gradient solvent system of 0.1% (*v*/*v*) TFA in water (solvent A) and 0.1% (*v*/*v*) TFA in CH_3_CN (solvent B) at a flow rate of 1.1 mL/min was used. The HPLC gradient began with a solvent composition of 95% A and 5% B from 0 to 2.5 min followed by a linear gradient of 95% A and 5% B to 5% A and 95% B over 30 min. The gradient remained at this position for 3 min before switching back to 95% A and 5% B for another 7 min. The main peak of each radiolabeled peptide was isolated, and acetonitrile then evaporated under a stream of nitrogen gas. The HPLC system (Shimadzu Corporation, Kyoto, Japan) was equipped with a UV-VIS detector (Shimadzu Corporation, Kyoto, Japan) set at 220 nm, a Flow-count γ-radioactivity detection system (Bioscan Inc., Washington, DC, USA) and the Lauralite HPLC chromatogram analysis program (LabLogic Systems Ltd., Sheffield, UK). Radiochemical purity was estimated by evaluating radioactivity peaks eluted for each ^99m^Tc-labeled peptide from the HPLC column and calculating the area under the peak (ROI). The HPLC-purified compounds were reconstituted in sterile saline and used for in vitro and in vivo experiments.

### 3.4. In Vitro Tumor Cell Binding and Cellular Internalization 

The cell-binding studies of ^99m^Tc-labeled HER2 and MUC1 peptides into respective HER2 and MUC1-positive human breast cancer cell lines (American Type Culture Collection, Rockville, MD, USA) was performed according to the method described previously [54]. In brief, ~300,000 cells (in 0.3 mL low-serum media) were incubated with various amounts of radiopeptides, ranging between 0.1 and 30 nM of peptides (prepared from the serial dilutions of HPLC-purified radiopeptides), in duplicate for 60 min at room temperature. Incubation was terminated by dilution with cold saline (0.3 mL) and the cells were pelleted by centrifugation. The cell-pellets was then rapidly washed with cold saline to remove any unbound peptide and centrifuged to collect supernatants. Radioactivity in the cell-pellet and the washings was measured in a γ-counter. Non-specific binding was determined in the presence of ~200-fold molar excess of unlabeled peptide. Specific binding was calculated by subtracting the non-specifically bound radioactivity from that of the total binding. The dissociation constant (*K*_d_) was calculated using a plot of specific cell-bound radioactivity versus increasing concentrations of the radiopeptide using a nonlinear regression analysis program (GraphPad Prism Software Inc., San Diego, CA, USA). 

In order to differentiate between cell-surface bound and cellular internalized radioactivity, cell pellet was treated with 0.3 mL of acidic buffer (0.02 M sodium acetate in saline, pH 5.0) for 10 min at 37 °C at the end of the binding experiment, followed by centrifugation and washing with cold acidic buffer. The amount of cell surface-bound (acid-wash) and internalized (acid-resistant) radioactivity was determined by measuring the radioactivity of the supernatant and the cell-pellet, respectively, in a γ-counter.

### 3.5. In Vivo Animal Biodistribution

Approval for the animal protocol used in this study was obtained from the Institutional Animal Care and Use Committee. Animal studies were conducted according to the international regulations governing the safe and proper use of laboratory animals [48]. In vivo biodistribution studies were performed on healthy Balb/c mice (n = 3–5 in each group, body mass 19–22 g) at 1 and 4 h after i.v. injection of the HPLC-purified radiopeptide (100 μL, 10–15 μCi, total peptide dose ~10 ng) via the lateral tail vein as described previously [54]. Mice were euthanized by cervical dislocation at specified times, the selected tissues/organs were removed, and radioactivity was measured in a γ-counter. Uptake of radioactivity in the tissues and organs was expressed as the percent injected dose per gram (% ID/g) of tissue/organ, which was calculated by comparison with standard solutions representing 10% of the injected dose per animal. For the clearance studies, radioactivity in the urine with bladder is expressed as the percent of the injected dose per organ (% ID/organ).

### 3.6. In Vivo Tumor Targeting and γ-Imaging

Approximately 7 million HER2-positive SKBR3 cells and MUC1-positive MCF7 and T47D breast tumor xenografts were injected subcutaneously into a group of nude female mice and allowed to grow for 4 to 6 weeks. After sufficient growth of tumors, the animals were sacrificed and the uptake of ^99m^Tc-HER2 and ^99m^Tc-MUC1 by the tumors and other major organs was determined by biodistribution experiments, as described above.

In general, for preclinical gamma camera imaging, each tumor-bearing mouse model was intravenously injected with ~200 μCi of ^99m^Tc-HER2 or ^99m^Tc-MUC1 peptide through the tail vein. Mouse static planar images were then acquired at 1 h after the radiotracer injection by a portable hand-held γ-camera equipped with CsI(Tl) scintillation detector, with intrinsic spatial resolution of 2.45 mm (IP guardian 2, Li-Tech, Italy). The injected peptide radiopharmaceutical usually travels through the body and gives off radiation in the form of gamma rays, which are detected by a gamma camera. The representations of the distribution of radiopharmaceuticals within the body was obtained in the form of readable medical images using sophisticated image reconstruction software.

### 3.7. Statistical Analysis

Results are expressed as mean ± S.D. where appropriate. For data comparisons, mean values were compared using the Student’s *t-*test (GraphPad Software, Inc., San Diego, CA, USA). A probability value (*p*) less than 0.05 was considered statistically significant.

## 4. Conclusions

Targeting tumor-associated antigens is an appealing approach for the diagnosis and treatment of solid and hematological malignancies. Tumor-associated antigens that are exclusively expressed by the tumor cells provide the ideal diagnostic and therapeutic target for molecular imaging. Molecular imaging has great potential to become a valuable tool in this new era of “personalized and precision medicine.” Important steps in adapting a new “image and treat” strategy in HER2-positive and MUC1-positive breast cancer have already been initiated. Along with more potential clinical studies and validation, molecular imaging of HER2-positive and MUC1-positive breast cancer could allow patient-customized dosage adaptation, with early finishing of ineffective and expensive therapies and reduction of “aggressive” treatment, ultimately resulting in cost savings, lower morbidity, and improved patient outcomes. Taken together, the importance of HER2 in cancer diagnosis/therapy is not only from its role in tumorigenesis, but also from its role as a maker for targeted delivery of various therapeutic agents.

In the present study, we performed in vitro and in vivo evaluation of HER2 and MUC1 derived peptides as potential candidates for breast cancer imaging. Our findings suggest that the tumor antigen peptides after radiolabeling with ^99m^Tc displayed good radiochemical and metabolic stability in vitro. The data from breast cancer cell binding studies confirmed the high affinity (low nanomolar range) towards respective breast cancer cell lines. In healthy mice, the ^99m^Tc-labeled peptides displayed favorable pharmacokinetics, with high excretion by the renal pathway. In tumor xenografts nude mice models, good uptake by the SKBR3, MCF7 and T47D tumors was found, with good tumor-to-blood and tumor to muscle ratios. Also tumor can be seen in γ-camera imaging. Although appropriate structural modification and more effort is required to enhance the tumor targeting characteristics of these peptides, the present work nonetheless showed the suitably of developing tumor-antigen derived peptides for imaging breast cancer. The knowledge gained from this study would give us the possibility to extend the scope of the study to other potential tumor-specific antigens peptides for better and effective diagnosis of human cancer.

## Figures and Tables

**Table 1:** Characterization of ^99m^Tc-HER2 and ^99m^Tc-MUC1 peptides.

	Molcular Weight *	% Labeling Efficiency	HPLC Retention Time (min)	Log P (Octanol/Saline)
^99m^Tc-HER2	2064	>95	20.0	1.38 ± 0.06
^99m^Tc-MUC1	972	>95	14.10	−2.22 ± 0.12

^*^ Molecular weight of unlabeled compounds.

**Table 2:** In vitro tumor cell-binding properties and cellular internalization of ^99m^Tc-HER2 and ^99m^Tc-MUC1 to respective receptor-positive breast cancer cell lines.

	Cell Line	*K*_d_ (nM)	% Internalization
^99m^Tc-HER2	SKBR3	49.97 ± 14.15	19.90 ± 5.35
	MCF7	157.92 ± 24.60	12.16 ± 3.20
	T47D	169.44 ± 22.29	11.85 ± 2.83
^99m^Tc-MUC1	MCF7	10.30 ± 1.55	18.92 ± 2.16
	T47D	3.48 ± 0.84	21.11 ± 2.95

## Abbreviations

TSA: Tumor-specific antigen; TAA: Tumor-associated antigen; MHC: Major histocompatibility; EGFR: The human epidermal growth factor receptor; PET: Positron-emission tomography; SPECT: Single-photon emission computed tomography; CT: Computed tomography; MRI: Magnetic resonance imaging; SPPS: Solid-phase peptide synthesis; ALA: Aminoleuvinic acid; *K*_d_: Dissociation constant; FDA: Food and Drug Administration; CTC: Circulating tumor cells; p.i.: Post-injection; ID: Injected dose; Glu: Glutamic acid; HYNIC: hydrazinonicotinamide; DTPA Diethylenetriaminepentaacetic acid; PEG: Polyethylene glycol; NOTA: 1,4,7-Triazacyclononane-1,4,7-triacetic acid; CB-TE2A: 1,4,8,11-Tetraazabicyclo[6.6.2]hexadecane-4,11-diacetic acid. DAP, Bis(3-dimethylarsinopropyl)phenyl-phosphine; DOX: Doxorubicin; PTX: Paclitaxel; mAb: Monoclonal antibody; ER: Estrogen-receptors; PgR: Progesterone-receptor.
